# Coloplasty Neorectum versus Straight Anastomosis in Low Rectal Cancers

**DOI:** 10.1155/2014/382371

**Published:** 2014-01-30

**Authors:** Fazl Q. Parray, Javaid A. Magray, Manzoor Ahmad Dar, Nisar A. Chowdri, Rauf A. Wani, Natasha Thakur

**Affiliations:** Colorectal Division, Department of General & Minimal Invasive Surgery, Sher-i-Kashmir Institute of Medical Sciences, Soura, Srinagar, J&K 190011, India

## Abstract

*Introduction*. Patients with the diagnosis of carcinoma rectum after random allocation were assigned to 2 groups. One group was subjected to total mesorectal excision with coloplasty neorectum reconstruction and another group to total mesorectal excision with straight anastomosis. This randomization was done by odds and even method by the sister in charge of the ward to avoid bias in randomization. The study included 42 patients with diagnosis of carcinoma rectum from 4 to 12 centimeters from anal verge. Composite incontinence score, bladder function, and sexual function were considered as the main outcome measures. *Results.* All patients of transverse coloplasty group had mild or moderate composite incontinence score while 7 (36.8%) patients of straight anastomosis group had a severe score at 7th POD (*P* < 0.05). At 6 months, 100% patients in transverse coloplasty group had a nil score which was not achieved by any of the patients in the other group. An intragroup comparison showed an improvement in score with time in both groups more marked in transverse coloplasty group. *Conclusion*. Transverse coloplasty group showed a better QOL so far as anal incontinence is considered. However, no statistically significant difference was achieved when comparing bladder and sexual dysfunction between the two groups.

## 1. Introduction

A better understanding of oncological factors governing tumor spread in rectal cancer, the advent of total mesorectal excision (TME) with nerve sparing, use of neoadjuvant chemoradiation, and the development of stapling devices have made it possible to avoid a permanent stoma in most of the patients undergoing surgery for low rectal carcinomas. A low anterior resection with restoration of bowel continuity is the surgical procedure of choice offered to such patients. However, performing a straight coloanal anastomosis for restoring the bowel continuity may be complicated by “anterior resection syndrome (ARS)” characterized by increase in defecatory frequency, urgency, and incontinence [1, 2]. This syndrome resulting from loss of rectal reservoir may affect up to 90% of patients with straight coloanal anastomosis and may worsen the quality of life in about 39% of patients [3].

Lazorthes and Parc developed the colonic J-pouch-anal and low rectal anastomosis in 1986 [4, 5], and its functional superiority over straight coloanal anastomosis was shown in randomized controlled trials [6–9]. However, 10–30% of patients with colonic J-pouch may experience some late evacuation problems with incomplete defecation requiring the use of laxatives, suppositories, and enemas [10–12]. This led surgeons to use smaller colon pouches (5-6 cm) [13] as against conventional larger colon pouches (8–10 cm) and the improved functional results with smaller pouches have been confirmed by randomized controlled trials [14, 15]. In addition, construction of a J-pouch can be technically difficult in a narrow male pelvis and in patients with a thick or short mesocolon [16].

To overcome these problems, a very small pouch, the transverse coloplasty pouch (TCP), was conceptualized by Z'graggen K and his colleagues and initially tested for its safety and early outcome in an animal model where it was compared with the standard operations like straight coloanal anastomosis and colon J-pouch [17, 18]. With the establishment of safety and excellent early results in animal model, the technique of transverse coloplasty pouch anastomosis was adapted in humans [17, 19]. It is claimed that this small volume reservoir, similar to pyloroplasty or strictureplasty, gives an improvement in early functional outcome and a decrease in late evacuation problems. The transverse coloplasty pouch is technically simpler than J-pouch and can be performed in presence of short or thick mesocolon or narrow male pelvis. Besides, TCP is more physiological and is in conformity with peristalsis at the time of defecation.

We undertook this study to compare the functional outcome and impact on overall quality of life in patients subjected to a straight coloanal anastomosis with those subjected to a transverse coloplasty pouch neorectum reconstruction.

## 2. Patients and Methods

The present prospective randomized study was conducted between June 2009 and October 2011 in which 42 patients with carcinoma rectum (4–12 centimeters) were included. The patients were randomly allocated to two groups to undergo straight or transverse coloplasty pouch anastomosis by odds and even method by the in charge staff nurse. One group of 22 patients was subjected to transverse coloplasty pouch (TCP) neorectum reconstruction and another group of 20 patients was subjected to straight coloanal anastomosis (SA) after low or ultralow anterior resection. The patients were diagnosed on the basis of clinical, endoscopic, and histopathological criteria. Specialized investigations like multidetector computed tomography, transrectal ultrasonography, or endorectal coil MRI were used for preoperative staging and assessment of operability. The clinical characteristics of patients are listed in Table 1.

Mechanical bowel preparation using Peglec or Colowash was given one day prior to surgery except in obstructing lesions. An informed consent for a temporary or permanent stoma was taken and stoma site was marked by stoma therapist with indelible ink prior to surgery. All patients underwent a standard low anterior resection with total mesorectal excision with preservation of nerves. Advantages and disadvantages of coloplasty were also explained in detail to the patients after randomization and a proper consent was taken for the procedure.

Descending colon after proper mobilization was used to make anastomosis in 16 (72.7%) patients of TCP group and sigmoid colon in the remainder whereas sigmoid colon was used in 13 (65.0%) patients of SA group to make the anastomosis and descending colon in the remainder. Articulating linear stapler or Access-55/Roticulator stapler was applied to anorectal junction to remove rectum and specimen. The anvil of circular stapler is inserted into the cut end of proximal colon and fixed with a purse string suture (1-0 prolene) tightened over it. About 5-6 cm from the cut end, an 8 cm longitudinal colostomy is performed between the taenia. Lateral traction by two-stay sutures applied forms a reservoir and the colostomy closed in a transverse fashion using 1-0 vicryl as is done in pyloroplasty. The anvil of the circular stapler was detached and fixed by a purse string suture of prolene in the distal end of colon. Circular stapler was introduced per anum and engaged with anvil. The stapler was fired to make the final anastomosis. The pelvis was filled with normal saline and air insufflated per anum blocking the colon proximal to anastomosis gently between fingers to check for any anastomotic leak. Ileostomy for temporary fecal diversion was used in 4 (18%) patients of TCP group and 3 (15%) of SA group.

Patients were discharged on 8th to 10th day and were advised to follow outpatient department at 15 days, 1 month, and 2 months and then at 6 monthly intervals. Functional outcome evaluation was done at 7th day, 2nd month, and 6th month focusing on the following points:

- frequency of bowel movements per 24 hours;
- nocturnal bowel movements;
- ability to defer defecation for more than 30 minutes;
- composite incontinence score;
- regular use of medication;
- ability to evacuate bowel within 15 minutes;
- sensation of incomplete evacuation.

The patients in whom a covering ileostomy/colostomy was done were evaluated for functional outcome only after their ostomies were closed and not after surgery.

## 3. Results

An *R*_0_ resection was achieved in most of the patients—90.9% of TCP group and 85% of SA group. One patient in TCP group died of postoperative sepsis and one patient in SA group died of pulmonary thromboembolism. Other postoperative complications are listed in Table 2.

Anastomotic stenosis was seen in SA group (21.1%) and was not seen in TCP group and the difference was statistically significant.

Frequency of bowel movements per 24 hours in the studied patients compared at 7th postoperative day (POD), 2 months, and 6 months is shown in Table 3.

No statistically significant difference was seen between two groups at 7th POD. However, at 2 months and 6 months, the mean number of bowel movements per 24 hours was 5 (3–6) and 2 (1–4) in TCP group and 10 (8–13) and 7 (5–9) in SA group thus achieving statistically a significant difference. An intragroup comparison also showed a statistically significant difference in bowel frequency with time more pronounced in TCP group.

A comparison of nocturnal bowel movements (NBM) was done between TCP and SA group at 7th POD, 2 months, and 6 months. At 2 months, 38.1% of patients in the TCP group and 89.5% of patients in SA group had NBM while no patient in TCP group at 6 months had an NBM compared to 84.2% of patients of SA group. An intragroup comparison showed significant improvement with time in NBM within TCP group only. The NBM frequency in studied patients is detailed in Table 4.

The ability to defer defecation for more than 30 minutes showed significantly better results in TCP group as compared to SA group at 7th POD, 2 months, and 6 months as detailed in Table 5. An intragroup comparison showed a significant improvement with time more in TCP group than in SA group.

A comparison of composite incontinence score (CIS) between two groups is tabulated in Table 6.

All patients of TCP group had mild or moderate score while 7 (36.8%) patients of SA group had a severe score at 7th POD (*P* < 0.05). At 2 months, 14 (66.75%) patients had a nil score while 2 (10.5%) patients of SA group continued to have a severe score (*P* < 0.05). At 6 months, 100% of patients in TCP group had a nil score which was not achieved by any of the patients of SA group. An intragroup comparison showed an improvement in CIS with time in both groups more marked in TCP group.

Quality of life (QOL) in the studied patients was assessed on the basis of

- anal incontinence,
- bladder dysfunction,
- sexual dysfunction.

Bladder dysfunction was assessed using International prostate symptom score (IPSS) and sexual dysfunction was assessed by international index of erectile function (IIEF) questionnaire and the results obtained are tabulated in Tables 7 and 8, respectively.

TCP group patients showed a better QOL in anal incontinence parameters. However, no statistically significant difference was achieved when comparing bladder and sexual dysfunction between the two groups.

## 4. Discussion

The modern day surgical treatment of rectal cancer started with abdominoperineal resection (APR) described by Czerny in 1884. The biggest disadvantage of APR is the creation of a permanent stoma with its associated psychosocial issues. With the development of circular staplers in the late 70's and early 80's, the trend changed towards sphincter saving resections (SSR) [20]. A further advance in the management of rectal cancer was made with the introduction of total mesorectal excision and the concept of 2 cm distal margin [21]. The current standard treatment for rectal cancer is a low anterior resection with total mesorectal excision with restoration of bowel continuity [21]. However, performing a straight coloanal anastomosis may be complicated by anterior resection syndrome as described earlier. In an attempt to improve functional results following low colorectal or coloanal anastomosis, Lazorthes and Parc in 1986 separately introduced the concept of constructing the colonic J-pouch [11, 16]. The standard colonic J-pouch achieved excellent early functional results [6], but up to 30% of patients experienced some late evacuation problems with incomplete defecation. This shifted the trend towards smaller J-pouches which significantly reduced the prevalence of evacuation problems from approximately 30% to 10% on long-term follow up [14, 15].

Z'graggen K and his colleagues in 2001 introduced a technically simpler transverse coloplasty pouch (TCP) [16], a novel pouch with a significantly smaller capacity than a colon J-pouch [17]. Z'graggen et al. in their study confirmed the safety of transverse coloplasty pouch for reconstruction after sphincter saving rectal resection. Their study showed a favorable early functional outcome following TCP with avoidance of late evacuation problems seen with colon pouch [22]. We, therefore, designed a study to compare the functional outcome and quality of life in patients who underwent a straight coloanal anastomosis with those who underwent a transverse coloplasty pouch reconstruction after tumor resection.

A complete *R*_0_ resection was achieved in 90.9% of patients of TCP group and 85% of SA group. Descending colon was used for making transverse coloplasty pouch in most of the patients rather than sigmoid colon as the later may have a fatty mesentery, present with diverticula, and show more propulsive motility than the descending colon. The rate of wound infection was 2 times more with SA group presumably because of low pre-op albumin levels. The rate of anastomotic leakage was similar with 20% in SA group and 18.2% in TCP group with an anastomotic structure found in 21.1% of patients of SA group and in none of the patients in TCP group. The higher incidence of anastomotic stricture in SA group can be due to low anastomosis (ultra LAR) or small size of the sample. One of the patients with anastomotic leak in TCP group required laparotomy for peritonitis. On exploration, it was found that the leak was on anterior wall of coloanal anastomosis below coloplasty. The remaining leaks were managed conservatively. Colonic J-pouch has decreased chances of anastomotic leak because of good blood supply across end to side anastomosis as against straight coloanal anastomosis [23]. Laser Doppler flow measurements in an antimesocolic transverse coloplasty do not show any impairment in perfusion proximal or distal to suture line [17]. Fistula formation was found in 2 (9.1%) of the TCP patients compared to none in the SA group which, however, was statistically insignificant.

The functional outcome following a straight coloanal anastomosis and J-pouch is related to the capacity of the neorectal reservoir [12, 24]. The TCP reduced the frequency of bowel movements when compared to straight coloanal anastomosis. The mean number of bowel movements per 24 hours at 2 months, followup in TCP group was 5 compared to 10 in SA group which was further reduced to 2 in TCP group and 7 in SA group at 6 months of followup which was significant and comparable to the results obtained after a colonic J-pouch procedure [6, 25]. Nocturnal bowel movements (NBM) were seen in 38.1% of the TCP group patients compared to 89.5% of patients of the SA group at 2 months follow-up and at 6 months none of the patients in TCP group had nocturnal bowel movement while 84.2% of SA group patients continued to have NBM which corresponded well with other studies [26].

Ability to defer defecation for more than 30 minutes was better in TCP group compared to SA group in our study with conflicting results from other studies.

In our study, retarding medications were used more frequently by SA group and bulking medications were sometimes used by TCP group which decreased with time but were never used by SA group with similar or better results reported by others [27, 28].

The ability to differentiate between gas and stool was significantly better in TCP group at all stages of followup with all of them being able to do so at 6 months while 57.9% of patients in SA group were still unable to differentiate between gas and stool.

The ability to evacuate bowel within 15 minutes was significantly better in SA group at 2 months. However, at 6 months followup, there was no significant difference between the two groups regarding bowel evacuation. TCP is an excellent choice to avoid late evacuation problems seen with colonic J-pouches as also supported by other studies [29, 30].

In our study, we found TCP group patients to be more continent to gases, liquids, and solids as compared to the SA group patients at 2 months and 6 months of followup. A composite incontinence scoring (CIS) was done in the study group. At 2 months, 66.75% of patients in the TCP group had nil CIS whereas 10.5% of patients had a severe CIS. At 6 months, all patients in TCP group had nil score while 26.3% of patients in SA group still had a moderate score. A similar improvement in the CIS was noted with time in both groups.

Quality of life (QOL) was assessed on the basis of anal incontinence, bladder dysfunction, and sexual dysfunction in the study group. TCP group showed a significant improvement in anal incontinence with time while bladder and sexual dysfunction showed no significant difference between the two groups.

## Figures and Tables

**Table 1:** Clinical characteristics.

Parameter	TCP group	SA group
Mean age (yr)	53.6 (range 22–80)	49.59 (range 19–75)
Male : female	1.4 : 1	1.2 : 1
Presenting symptoms		
Bleeding P/R	22 (100%)	20 (100%)
Constipation	14 (63.6%)	10 (50%)
Tenesmus	12 (54.5%)	10 (50%)
Diarrhoea	12 (54.5%)	5 (25%)
Urgency	5 (22.7%)	10 (50%)
Night soiling	4 (18.2%)	3 (15%)
Mean duration of symptoms (months)	4.1 (range 2–8)	4.5 (range 2–8)
Tumor location (mean distance in cm from anal verge)	7.6 (range 5–12)	7.3 (range 5–12)
Dukes stage of tumor (number of pts.)		
*A*	9 (40.9%)	7 (35%)
*B*	4 (18.2%)	2 (10%)
*C*_1_	8 (36.4%)	9 (45%)
*C*_2_	1 (4.5%)	2 (10%)

**Table 2:** Postoperative complications.

	TCP group		SA group		*P* value
	*N*	%	*N*	%	
Anastomotic leak	4	18.2	4	20	0.882 (NS)
Wound infection	5	22.7	9	45	0.131 (NS)
Pneumonia	5	22.7	7	35	0.385 (NS)
Fistula	2	9.1	0	0	0.172 (NS)
Urinary retention	3	13.6	0	0	0.090 (NS)
Anastomotic stenosis	0	0	4	21.1	0.025 (Sig.)
Intestinal obstruction	0	0	0	0	1.000 (NS)
DVT	1	4.5	0	0	0.353 (NS)

**Table 3:** Frequency of bowel movements per 24 hours.

	TCP group		SA group		Between group comparison
	*N*	%	*N*	%	*P* value
7th day					
≤2	18	85.7	12	63.2	0.104 (NS)
>2	3	14.3	7	36.8	
Median	2 (1–3)		2 (1–3)	
2nd month					
≤2	0	0	0	0	1.000 (NS)
>2	21	100	19	100	
Median	5 (3–6)		10 (8–13)	
6th month					
≤2	15	71.4	0	0	0.000 (Sig)
>2	6	28.6	19	100	
Median	2 (1–4)		7 (5–9)	
Within group comparison					
*P* value	0.000 (Sig)		0.000 (Sig)		

**Table 4:** Nocturnal bowel movements (NBM).

	TCP group		SA group		*P* value
	*N*	%	*N*	%	
7th day	12	57.1	15	78.9	0.147 (NS)
2nd month	8	38.1	17	89.5	0.001 (Sig.)
6th month	0	0.0	16	84.2	0.000 (Sig.)
Within group comparison (*P* value)	0.003 (Sig.)		0.651 (NS)		

**Table 5:** Ability to defer defecation for >30 minutes.

	TCP group		SA group		*P* value
	*N*	%	*N*	%	
7th day					
Never	15	71.4	19	100	0.013 (Sig.)
Sometimes	6	28.6	0	0.0	
Often	0	0.0	0	0.0	
Always	0	0.0	0	0.0	
2nd month					
Never	0	0.0	12	63.2	0.000 (Sig.)
Sometimes	10	47.6	7	36.8	
Often	9	42.9	0	0.0	
Always	2	9.5	0	0.0	
6th month					
Never	0	0.0	6	31.6	0.000 (Sig.)
Sometimes	0	0.0	13	68.4	
Often	13	61.9	0	0.0	
Always	8	38.1	0	0.0	
Within group comparison					
*P* value	0.000 (Sig.)		0.001 (Sig.)		

**Table 6:** Composite incontinence score.

	TCP group		SA group		*P* value
	*N*	%	*N*	%	
7th day					
Nil	0	0.0	0	0.0	0.001 (Sig.)
Mild	2	9.5	0	0.0	
Moderate	19	90.5	12	63.2	
Severe	0	0.0	7	36.8	
2nd month					
Nil	14	66.7	0	0.0	0.000 (Sig)
Mild	6	28.6	4	21.1	
Moderate	1	4.8	13	68.4	
Severe	0	0.0	2	10.5	
6th month					
Nil	21	100.0	0	0.0	0.000 (Sig.)
Mild	0	0.0	14	73.7	
Moderate	0	0.0	5	26.3	
Severe	0	0.0	0	0.0	
Within group comparison					
*P* value	0.000 (Sig.)		0.000 (Sig.)		

**Table 7:** Bladder dysfunction.

	TCP group		SA group		*P* Value
	*N*	%	*N*	%	
Pre-op					
Nil	21	95.5	20	100.0	0.340 (NS)
Mild	1	4.5	0	0.0	
2nd month					
Nil	19	90.5	17	89.5	0.917 (NS)
Mild	2	9.5	2	10.5	
6th month					
Nil	20	95.2	17	89.5	0.495 (NS)
Mild	1	4.8	2	10.5	
Within group comparison					
*P* value	0.368 (NS)		0.135 (NS)		

Pre-op: Pre-operative.

**Table 8:** Sexual dysfunction.

	TCP group		SA group		*P* value
	*N*	%	*N*	%	
Pre-op					
No dysfunction	22	100.0	20	100.0	1.000 (NS)
Mild dysfunction	0	0.0	0	0.0	
2nd month					
No dysfunction	17	81.0	16	84.2	0.789 (NS)
Mild dysfunction	4	19.0	3	15.8	
6th month					
No dysfunction	18	85.7	16	84.2	0.896 (NS)
Mild dysfunction	3	14.3	3	15.8	
Within group comparison					
*P* value	0.039 (NS)		0.050 (NS)		

Pre-op: Pre-operative.
